# *Bacillus subtilis* SepF Binds to the C-Terminus of FtsZ

**DOI:** 10.1371/journal.pone.0043293

**Published:** 2012-08-13

**Authors:** Ewa Cendrowicz, Sebastiaan P. van Kessel, Laura S. van Bezouwen, Neeraj Kumar, Egbert J. Boekema, Dirk-Jan Scheffers

**Affiliations:** 1 Department of Molecular Microbiology, Groningen Biomolecular Sciences and Biotechnology Institute, University of Groningen, Groningen, The Netherlands; 2 Department of Electron Microscopy, Groningen Biomolecular Sciences and Biotechnology Institute, University of Groningen, Groningen, The Netherlands; Institut Pasteur Paris, France

## Abstract

Bacterial cell division is mediated by a multi-protein machine known as the “divisome”, which assembles at the site of cell division. Formation of the divisome starts with the polymerization of the tubulin-like protein FtsZ into a ring, the Z-ring. Z-ring formation is under tight control to ensure bacteria divide at the right time and place. Several proteins bind to the Z-ring to mediate its membrane association and persistence throughout the division process. A conserved stretch of amino acids at the C-terminus of FtsZ appears to be involved in many interactions with other proteins. Here, we describe a novel pull-down assay to look for binding partners of the FtsZ C-terminus, using a HaloTag affinity tag fused to the C-terminal 69 amino acids of *B. subtilis* FtsZ. Using lysates of *Escherichia coli* overexpressing several *B. subtilis* cell division proteins as prey we show that the FtsZ C-terminus specifically pulls down SepF, but not EzrA or MinC, and that the interaction depends on a conserved 16 amino acid stretch at the extreme C-terminus. In a reverse pull-down SepF binds to full-length FtsZ but not to a FtsZΔC16 truncate or FtsZ with a mutation of a conserved proline in the C-terminus. We show that the FtsZ C-terminus is required for the formation of tubules from FtsZ polymers by SepF rings. An alanine-scan of the conserved 16 amino acid stretch shows that many mutations affect SepF binding. Combined with the observation that SepF also interacts with the C-terminus of *E. coli* FtsZ, which is not an *in vivo* binding partner, we propose that the secondary and tertiary structure of the FtsZ C-terminus, rather than specific amino acids, are recognized by SepF.

## Introduction

Bacterial cell division is mediated by a multi-protein machine known as the “divisome” that assembles into a ring at the site of cell division [1]. Formation of the divisome starts with the polymerization of the tubulin-like protein FtsZ into a ring, the Z-ring, just below the membrane at the cell division site. Because of its critical role in division, Z-ring formation is under tight control to ensure bacteria divide at the right time and place. Once formed, the Z-ring functions as a scaffold to which all other division proteins are recruited, and several proteins bind to the Z-ring to mediate its membrane association and persistence throughout the division process [2].

Placement of the Z-ring is mediated by two systems, that prevent cells from dividing at the poles or immediately after division is completed (Min), or from dividing through nucleoids (nucleoid occlusion). The MinC component of Min directly acts on FtsZ [3], [4], whereas nucleoid occlusion is mediated by the FtsZ-binding protein SlmA in *E. coli* [5], [6] and the non-homologous protein Noc in *B. subtilis,* which does not seem to act directly on FtsZ [7]. In *B. subtilis*, Z-ring formation is also controlled by the nutritional status of the cell, through the nutritional sensor UgtP that acts directly on FtsZ [8]. Another level of control is provided by ClpX. In *B. subtilis* ClpX destabilizes, but not degrades FtsZ polymers, whereas in *E. coli* ClpX degrades (preferentially polymeric) FtsZ and thus controls the amount of FtsZ available for Z-ring formation [9], [10].

The Z-ring is tethered to the membrane by FtsA. In *E. coli*, ZipA forms an additional membrane tethering factor [11]. A protein with a similar topology to ZipA, EzrA, acts as a destabilizer of Z-rings in *B. subtilis* [12]. Z-rings are further stabilized by ZapA [13], a protein that strengthens lateral associations between FtsZ filaments, and in a small subset of bacteria including *E. coli,* ZapC fulfills a similar function [14], [15]. Other proteins that interact directly with FtsZ have a specific function in division. FtsE functions as part of the FtsEX complex that controls hydrolysis of the cell wall in dividing *E. coli* [16], [17]. In *B. subtilis,* SepF is a ring-forming protein that binds FtsZ and plays a role in correct assembly of the cross-wall septum, presumably by bundling FtsZ polymers into ∼50 nm wide tubes that can be observed *in vitro* [18]. During sporulation, SpoIIE interacts with FtsZ to ensure proper formation of the asymmetric septum [19] and MciZ, a 40 amino acid protein, prevents aberrant FtsZ ring formation in the mother cell compartment [20].

Even though crystal structures of FtsZ and various interacting proteins exist, relatively little is known about the precise interactions between FtsZ and these proteins. However, it is increasingly evident that a conserved stretch of amino acids at the C-terminus of FtsZ is involved in many of these interactions. The high degree of conservation of this C-terminal peptide was identified by Ma and Margolin [21]. The C-terminal peptide consists of a highly conserved core sequence followed by a variable number of poorly conserved residues at the extreme C-terminus. This region is not resolved in the available FtsZ structures, as it is preceded by a non-conserved, flexible linker sequence of variable length. The FtsZ-binding domain of ZipA has been co-crystallized together with a peptide matching the FtsZ C-terminus, which showed an extended β-strand followed by an α-helix [22]. In addition to ZipA, evidence has been reported for the interaction of the conserved FtsZ C-terminus with MinC [23], FtsA [24], EzrA [25], ClpX [9], SepF [26] and FtsZ itself [27]. Evidence for the interaction of FtsA with the C-terminus is based on yeast two-hybrid data [24]. For MinC, mutations in the FtsZ C-terminus conserved core have been described that abolish the interaction with MinC both *in vivo* and *in vitro* [23]. Using a C-terminal truncation of FtsZ (FtsZ_Ec_Δ18) that is still capable of polymerization, it was shown that ClpX is far less active in degradation of FtsZ_Ec_Δ18 than of wt FtsZ [9]. All these studies were done in *E. coli*. In *Mycobacterium tuberculosis*, the FtsZ C-terminus mediates the interaction between FtsZ and FtsW [28]. EzrA and SepF are Gram-positive division proteins that modulate FtsZ. The effects of EzrA or SepF on FtsZ polymerization can be relieved by adding competing amounts of a synthetic peptide encoding the FtsZ C-terminus, and EzrA and SepF have no effects on a C-terminal truncation mutant of FtsZ that still polymerizes (FtsZ_Bs_Δ16) [25], [26]. The SepF study showed that SepF bundles FtsZ filaments and co-sediments with FtsZ in polymerization experiments. The FtsZ_Bs_Δ16 truncate still polymerized but no longer bundled in the presence of SepF, yet SepF still co-sedimented with FtsZ_Bs_Δ16, leading the authors to suggest that SepF also binds to a secondary site on FtsZ next to the C-terminus [26]. Recently the poorly conserved residues at the extreme C-terminus have been implicated in lateral interactions between FtsZ polymers that are potentially mediated by electrostatic forces [27].

The crystal structure of the FtsZ-binding domain of ZipA bound to a peptide matching the FtsZ C-terminus [22], and the recent crystal and NMR structures on the interaction of *Thermotoga maritima* FtsA (FtsA_Tm_) and a FtsZ_Tm_ C-terminal peptide [29] provide more insight in how the C-terminus is bound by proteins. Bound to ZipA, the C-terminal peptide assumes the confirmation of an extended β-strand followed by an α-helix, whereas bound to FtsA the peptide is predominantly helical, but not as a single helix. Six residues in the peptide interact with ZipA, mostly through hydrophobic contacts but also through two hydrogen bonds between the backbones of ZipA and the FtsZ-peptide, formed by a conserved Asp and Leu residues (D367 and L369 in *B. subtilis* FtsZ) [22]. The interactions with FtsA are quite different, with three salt bridges connecting the peptide to FtsA. The salt bridges are formed by a conserved Asp and Arg residue (D370 and L376 in *B. subtilis* FtsZ) and the carboxyl group of the C-terminal amino acid residue [29]. Based on a comparison of both structures it has been suggested that the FtsZ C-terminus can adopt different conformations to fit different binding pockets [29].

In this study, we describe a pull-down strategy to identify interaction partners of the *B. subtilis* FtsZ C-terminus. Using this strategy we identify SepF as a binding partner of the C-terminus, confirming an earlier observation of Singh *et* *al*. [26]. We extend the earlier results by showing that SepF only binds to the C-terminus and by identifying residues critical for binding of SepF by an alanine-scan of the FtsZ C-terminus.

## Results

### SepF binds to the FtsZ C-terminus

**Figure 1:**
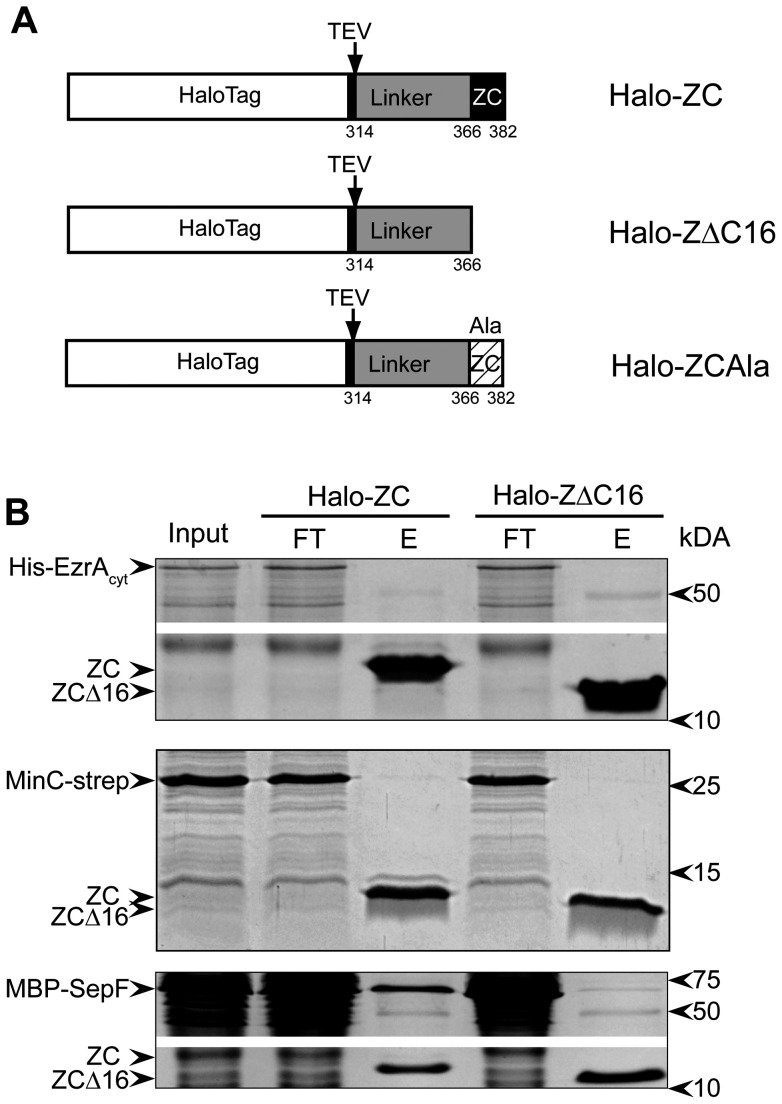
MBP-SepF binds to the FtsZ C-terminus. (A) Schematic representation of the Halo-Tagged constructs used for the pull-down experiments. Halo-ZC consists of the HaloTag followed by a TEV protease cleavage site fused to the final 69 amino acids of *B. subtilis* FtsZ, including the 16 amino acid residues of the conserved C-terminal tail. Halo-ZΔC16 contains only the 53 amino acid residues of the FtsZ linker-domain but lacks the conserved C-terminal tail and Halo-Z_Ala_ is representative of the constructs used for the alanine scanning of the conserved C-terminal tail. (B) Pull down experiments using Halo-Zc or Halo ZΔC16 bound to resin. Pull downs were performed on lysates from *E. coli* cells overexpressing His-EzrA_cyt_ (top panel), MinC-strep (middle panel) and MBP-SepF (lower panel). The input material (Input), flow-through (FT) and eluate (E) fractions of the experiments are shown and relevant proteins are indicated on the left. In the top and lower panel a fragment of the gel that did not contain the bait proteins or the protein of interest was deleted (white bar) to reduce the size of the image.

To screen for *B. subtilis* proteins that interact with the FtsZ C-terminus we fused the last 69 amino-acid residues of *B. subtilis* FtsZ to a HaloTag (Halo-ZC, Fig. 1A). The HaloTag consists of a modified dehalogenase that can covalently bind to a chloroalkane ligand coupled to sepharose beads (HaloLink resin) [30]. As a control we fused only the flexible, non-conserved linker sequence of FtsZ without the final 16 amino acid residues to a HaloTag (Halo-ZΔC16). The fusion proteins were expressed in *E. coli* and bound to the Halolink resin in binding buffer. Aliquots of the resin containing the covalently attached bait protein were subsequently used for pull-down experiments.

To validate the pull-down approach we analyzed the binding of three candidate interacting proteins after overexpression in *E. coli*. EzrA and SepF have been reported to interact with the FtsZ C-terminus in *B. subtilis*, and MinC interacts with the FtsZ C-terminus in *E. coli* [23], [25], [26]. We expressed *B. subtilis* MinC fused to a strep-tag (MinC-strep), the cytosolic domain of EzrA fused to a His-tag (His-EzrA_cyt_), and SepF fused to Maltose-Binding protein (MBP-SepF) in *E. coli* cells, prepared cell lysates and incubated the lysates with aliquots of resin with attached bait protein. After extensive washing, the bait protein and any interacting proteins bound to it were eluted from the resin by TEV protease cleavage. We found that MBP-SepF was retained by Halo-ZC bound to resin, and very weakly by Halo-ZΔC16 bound to resin (Fig. 1B). This confirms an earlier report that suggested that SepF binds to the FtsZ C-terminus [26]. His-EzrA_cyt_ and MinC-strep were not retained by either construct, also not when we tested for the presence of prey protein using specific antibodies against the His- or strep-tag (not shown). Previously, we had not been able to establish a direct interaction between *B. subtilis* MinC and FtsZ [31].

### The interaction between the FtsZ C-terminus and SepF is specific

**Figure 2:**
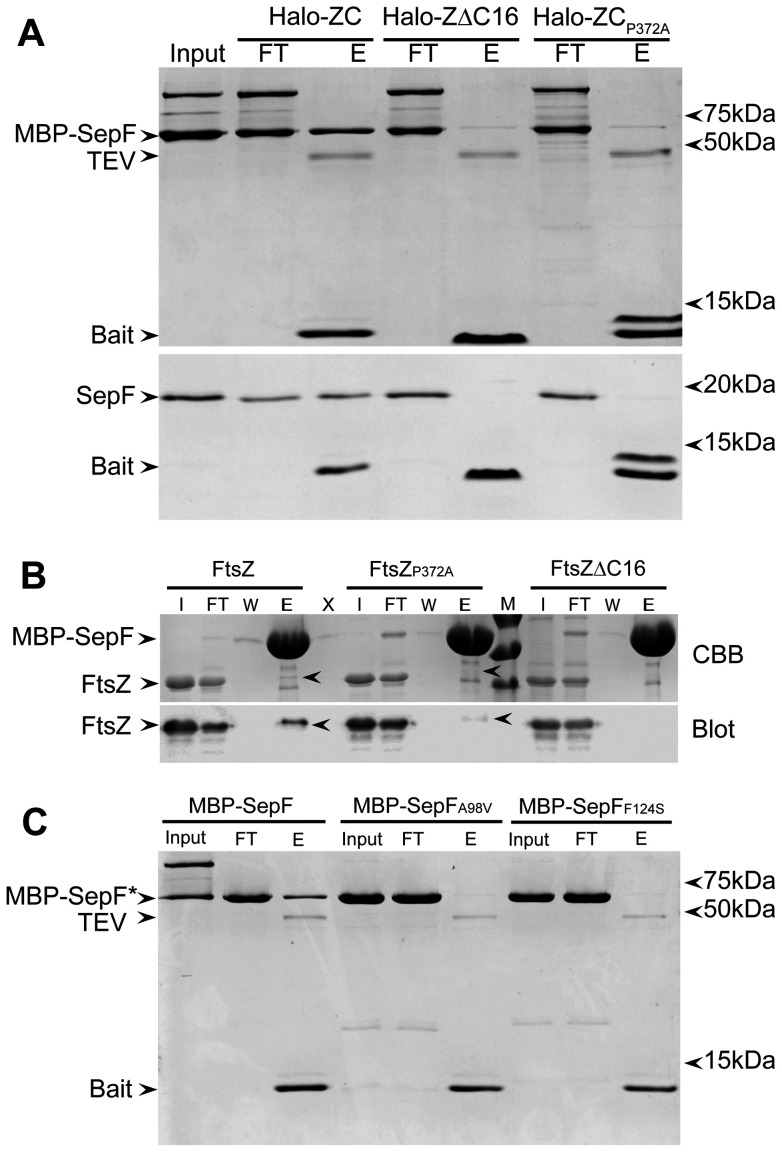
Specificity of the interaction between SepF and the FtsZ C-terminus. (A) Pull down experiments using Halo-ZC, Halo ZΔC16 or Halo-ZC_P372A_ bound to resin, with MBP-SepF (upper panel) or wild type SepF (lower panel) as prey proteins. The input material (Input), flow-through (FT) and eluate (E) fractions of the experiments are shown and relevant proteins are indicated on the left. MBP-SepF tends to form dimers as can be observed from the extra band in the input and flow-through samples. (B) Reverse pull down experiment with MBP-SepF bound to resin and purified FtsZ, FtsZ_P372A_ or FtsZΔC16 used as prey proteins. The input material (I), flow-through (FT), last wash (W) and eluate (E) fractions of the experiments are shown and relevant proteins are indicated on the left. M: molecular weight marker, bands are 70, 55 and 40 kDa. Upper panel: Coomassie stained gel (CBB), lower panel: western blot developed with anti-FtsZ antibody. (C) Pull down experiments using Halo-Zc bound to resin, with MBP-SepF, MBP-SepF_A98V_ and MBP-SepF_F124S_ as prey proteins. The input material (Input), flow-through (FT) and eluate (E) fractions of the experiments are shown and relevant proteins are indicated on the left.

To study the interaction between SepF and the FtsZ C-terminus in more detail, and to exclude that the interaction is mediated by potential ‘bridging’ proteins present in the *E. coli* lysate, we repeated the pull-down experiments with purified MBP-SepF and SepF. As an extra control, we used a bait construct in which a conserved proline residue in the C-terminus was mutated into alanine, Halo-ZC_P372A_. Pro372 (notation for full length FtsZ) is fully conserved in the FtsZ C-terminus across species and we reasoned that a mutation of this residue would abolish the capability of the C-terminus to interact with SepF. Both pure MBP-SepF and SepF were retained when Halo-ZC was used as bait protein. When Halo-ZΔC16 or Halo-ZC_P372A_ were used as bait a very faint band of MBP-SepF was visible in the eluate, but untagged SepF was not retained (Fig. 2A). This confirms that the interaction between SepF and the FtsZ C-terminus is specific, and that the pull-down is not caused by aspecific binding of the MBP-moiety to the C-terminus. The result also shows that the conserved proline residue indeed is critical for the function of the FtsZ C-terminus. We noticed that the ZC_P372A_ bait protein is visible in the eluate as two bands on gel. The protein is initially expressed as full-length protein, during elution the ZC_P372A_ bait is released from the Halo-tag by TEV cleavage. The double band pattern can be attributed to a change in the electrophoretic pattern of the mutated bait protein which is not completely resolved by the sample buffer resulting in two bands. Another, less likely explanation is that the alanine substitution gives rise to an extra TEV cleavage site. Other alanine substitutions in the C-terminus also give rise to double bands for the bait protein, and in several cases this change did not affect SepF binding (see below).

MBP-SepF bound to amylose resin can be used to pull down FtsZ from *E. coli* lysates expressing *B. subtilis* FtsZ [18]. This allowed us to perform the reverse pull-down experiment, and we incubated MBP-SepF bound to resin with purified FtsZ, FtsZΔC16 and FtsZ_P372A_. Importantly, in this experiment full-length FtsZ was used, so if there is a secondary binding site for SepF on FtsZ, one could expect that all three prey proteins are pulled down by MBP-SepF. We found that wild type FtsZ was bound by MBP-SepF and could be detected in the eluted fraction both by Coomassie staining and by western blotting (Fig. 2B). FtsZΔC16 and FtsZ_P372A_ were not visibly co-eluted with MBP-SepF with Coomassie, and only a very faint band was detected for co-eluted FtsZ_P372A_. This result suggests that the FtsZ C-terminus is either the only site of interaction with SepF, or that the binding of SepF to the secondary binding site is weak.

Two mutations in SepF, SepF_A98V_ and SepF_F124S_ have been described that abolish SepF-FtsZ interaction [18]. We purified MBP-SepF_A98V_ and MBP-SepF_F124S_ and used these proteins as prey in a Halo-ZC pull-down. Neither SepF mutant was found to bind to the FtsZ C-terminus (Fig. 2C). This suggests that the mutations in SepF_A98V_ and SepF_F124S_ abolish binding of SepF to the FtsZ C-terminus rather than to a secondary binding site on FtsZ. It is also of note that whereas MBP-SepF runs as monomers and dimers on SDS-PAGE, the two mutants do not, confirming the observation that both mutants, most notably SepF_A98V_, are less prone to form large oligomeric structures [18].

Taken together, these results show that SepF interacts with the FtsZ C-terminus, that this interaction is specific, and that we cannot find evidence for a secondary interaction site on FtsZ using pull-down assays.

### SepF cannot tubulate FtsZΔC16 and FtsZ_P372A_ polymers

**Figure 3:**
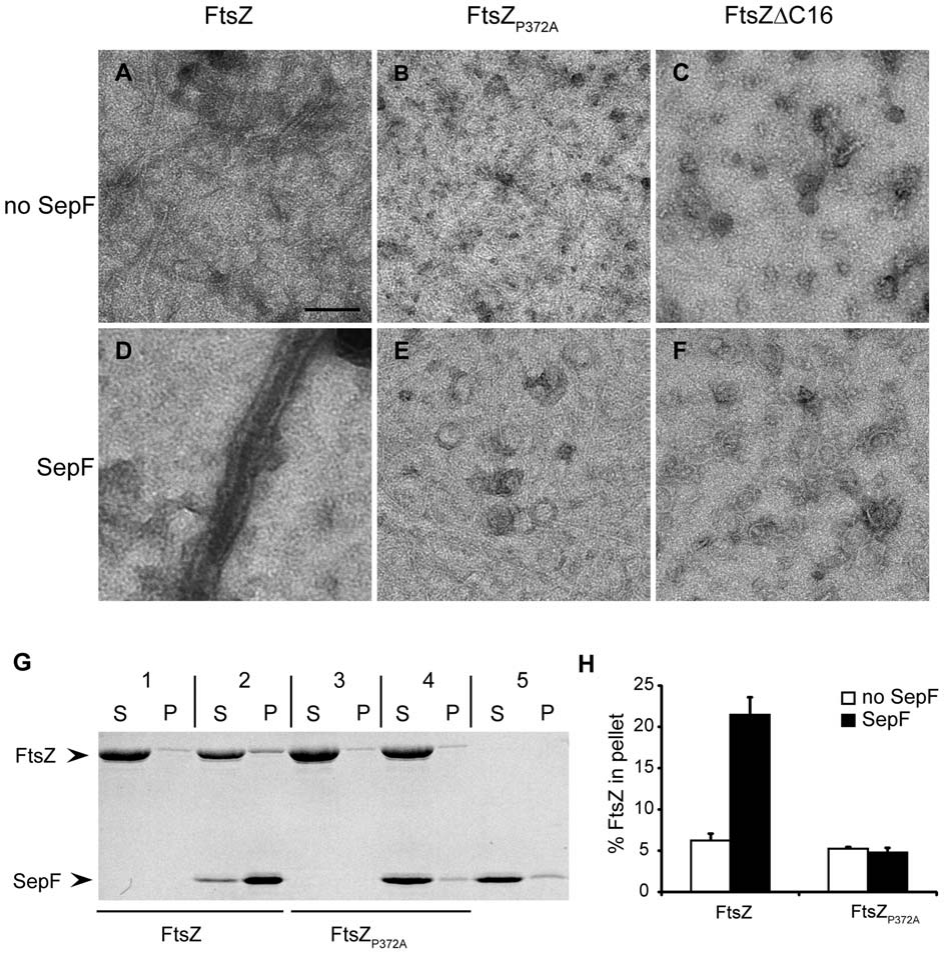
SepF cannot tubulate FtsZΔC16 and FtsZ_P372A_ polymers. (A–F) Electron microscopy of 10 μM FtsZ (A, D), FtsZ_ P372A_ (B, E) and FtsZΔC16 (C, F) polymerized with 2 mM GTP in the absence (A–C) or presence (D–F) of 6 μM SepF. Scale bar: 100 nm. (G) Pelleting of FtsZ-SepF tubules by centrifugation. 10 μM FtsZ (1, 2) or FtsZ_ P372A_ (3,4) was polymerized with 2 mM GTP in the absence (1,3) or presence (2,4) of 12 μM SepF. As a control, SepF was used without FtsZ (5). Tubules were recovered by centrifugation and supernatant and pellet fractions were analyzed by SDS-PAGE. (H) Quantification of the amount of FtsZ pelleted in the assay shown in (G). Three independent experiments were analyzed, error bars indicate standard deviation.

*In vitro*, SepF rings and FtsZ polymers combine to form large tubular structures [18]. Our pull-down experiments show that FtsZΔC16 and FtsZ_P372A_ have lost the ability to bind to SepF, but we cannot exclude that a weak residual interaction between the proteins (*e.g.* through a secondary interaction site on FtsZ) still allows the formation of tubules. Therefore, we mixed FtsZ, FtsZ_P372A_ and FtsZΔC16 with SepF and GTP, to induce FtsZ polymerization and possible tubule formation, and examined the samples using electron microscopy. FtsZ formed tubules with SepF (Fig. 3D), but in the case of FtsZ_P372A_ and FtsZΔC16 we could detect both FtsZ polymers and SepF rings (Fig. 3E, F), but not tubules, despite careful examination of the sample grids. We noticed that under our experimental conditions, FtsZ_P372A_ readily polymerized, but that FtsZΔC16 only formed very short polymers and aggregates (Fig. 3A–C), unlike the single stranded filaments that were reported for a FtsZΔC17 mutant [27]. We attribute this difference to the buffer system used in our study, with an increased pH and salt concentration that has previously been reported to decrease the amount of FtsZ polymerized in centrifugation and light scattering assays [18].

As a second assay for tubule formation we used sedimentation, at a velocity whereby SepF-FtsZ tubules but not FtsZ polymers are recovered in the pellet. FtsZ and FtsZ_P372A_ were polymerized with GTP in the presence and absence of SepF. FtsZΔC16 was not used in this assay because of its tendency to aggregate. FtsZ was recovered in the pellet above background levels only when SepF was present, whereas FtsZ_P372A_ was not pelleted with SepF (Fig. 3G, H). SepF sedimentation is dependent on the presence of FtsZ, and does not occur with FtsZ_P372A_, or when SepF is incubated alone (Fig. 3G). Combined with the pull-down experiments above this provides evidence that the FtsZ C-terminus is the only site of interaction with SepF.

### The SepF binding site arises from the folding of the FtsZ C-terminus

**Figure 4:**
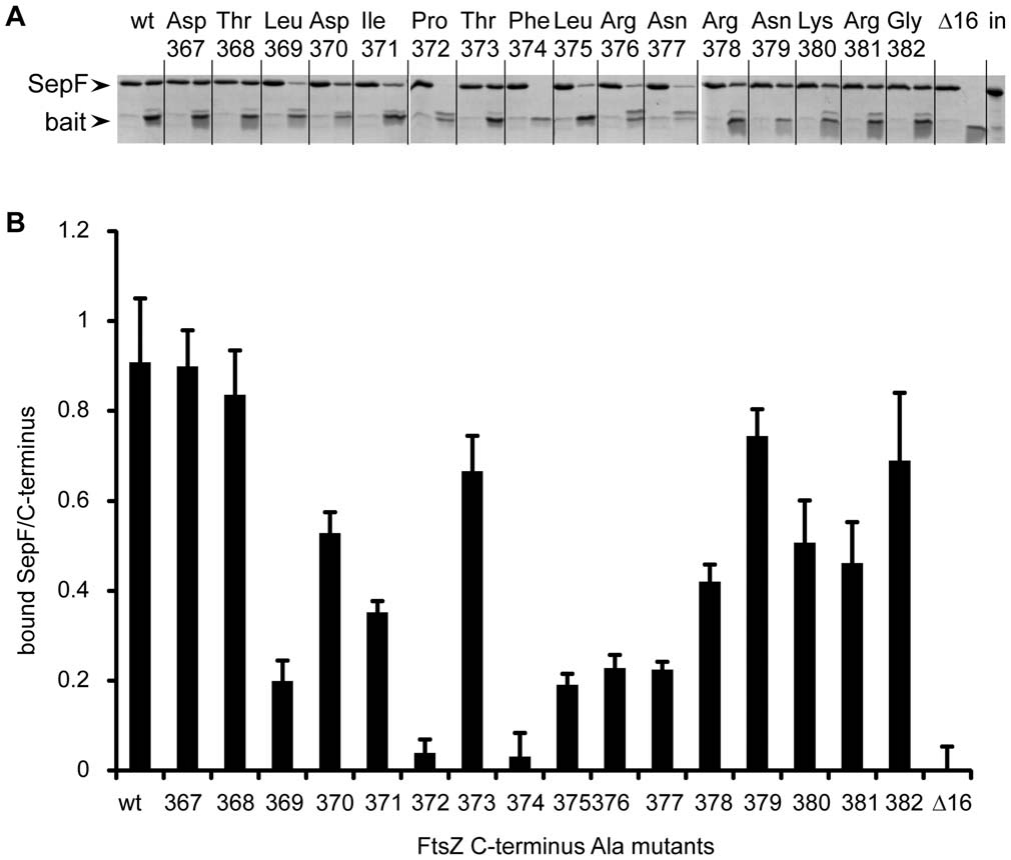
Alanine scanning of the FtsZ C-terminus. (A) Series of three gels from a representative pull down experiment using Halo-ZC (wt), Halo ZΔC16 (Δ16) and a series of HaloZC_Ala_ constructs (mutated residue indicated). For each pull down the flow-through (left side) and eluate fractions (right side) were loaded on the gel. The last lane is a loading control containing an equivalent amount of input SepF material. (B) Ratio of eluted SepF over eluted bait protein as determined by densitometric scanning of gels as shown in (A). Three independent experiments were analyzed, error bars indicate standard deviation.

The Halo-ZC construct specifically pulls down SepF, and this interaction is lost upon deletion of the final 16 residues from the C-terminus or by mutating the conserved Pro372 to Ala. To learn which other residues in the conserved C-terminus are critical for SepF binding, we performed an alanine-scan of the C-terminus. Each of the last 16 residues of the C-terminus was mutated to Ala (Fig. 1A), resulting in a series of HaloZC_Ala_ constructs which were used to pull down purified SepF (Fig. 4A). We determined the intensity of Coomassie-stained bands from the eluted C-terminus and the co-eluted SepF, and plotted the ratio of the densities as an indication for binding, using Halo-ZC and Halo-ZΔC16 as references (Fig. 4B). The use of ratios of bound SepF to eluted bait protein means that we have corrected for possible fluctuations in the amount of the bait protein bound to and/or eluted from the beads. Changing the fully conserved Pro372 and Phe374 residues abolished SepF binding. Changing Leu369, Leu375, Arg376 and Asn377 reduced SepF binding to less than 25% of the binding observed with the wild type. Changing Asp370, Ile371, Arg378, Lys380 and Arg381 also notably affected SepF binding, whereas changing Asp367, Thr368, Thr373, Asn379 and Gly382 resulted in SepF recovery that was not noticeably different from wild type. The fact that 11 out of 16 mutations inhibited SepF binding to various extent, suggests that the recognition of the FtsZ C-terminus by SepF depends more on the folding of the C-terminus than on specific interactions between amino acids in the form of salt bridges.

**Figure 5:**
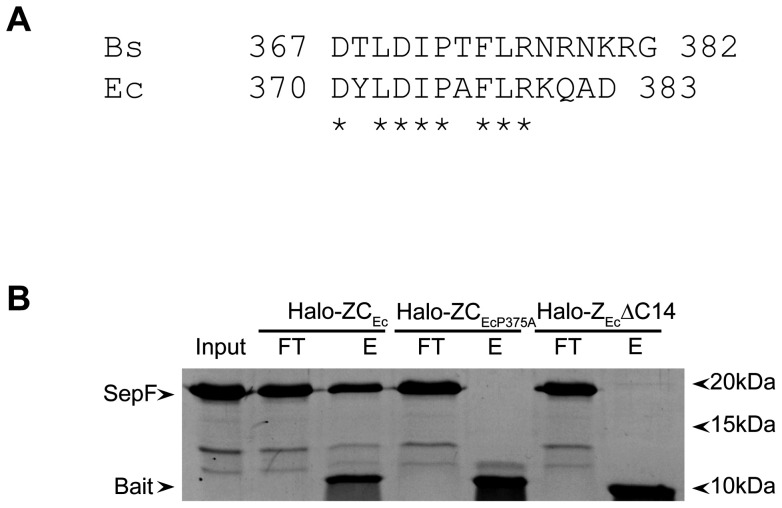
SepF binds to the C-terminus of E. coli FtsZ. (A) Alignment of the C-termini of *B. subtilis* (Bs) and *E. coli* (Ec) FtsZ. Identical residues are marked with an asterisk. (B) Pull down experiments using Halo-ZCEc, Halo-ZC_EcP372A_ or Halo-Z_Ec_ΔC14 bound to resin, with wild type SepF as prey protein. The input material (Input), flow-through (FT) and eluate (E) fractions of the experiment are shown and relevant proteins are indicated on the left.

The *E. coli* FtsZ C-terminus has a conserved part that is very similar to that of *B. subtilis* FtsZ (Fig. 5A), yet as *E. coli* does not have SepF there is no need for FtsZ_Ec_ to bind SepF. We reasoned that if the overall conformation of the C-terminus is recognized by SepF, then SepF might recognize the *E. coli* FtsZ C-terminus as well. We constructed Halo-ZC_Ec_, Halo-Z_Ec_ΔC14 and Halo-ZC_EcP375A_ similar to our *B. subtilis* constructs and performed a pull-down with purified SepF (Fig. 5B). SepF was bound to the Halo-ZC_Ec_ construct but not by Halo-Z_Ec_ΔC14 or Halo-ZC_EcP375A_. This result shows that SepF is capable of recognizing the C-terminus of a homologous FtsZ molecule, although this homologous FtsZ is not a binding partner for SepF *in vivo*.

## Discussion

In this paper we describe a novel pull-down assay to detect protein-protein interactions of the FtsZ C-terminus. The assay is based on the HaloTag fusion technology in which the bait protein is covalently bound to a resin allowing stringent wash steps before applying a sample to the resin [30]. Our fusion constructs consist of a modified dehalogenase fused to a part of FtsZ containing both the long flexible, non-conserved linker sequence and the conserved FtsZ C-terminus. The presence of the linker sequence has a dual function, it provides flexibility and distances the C-terminus from the dehalogenase, and it provides a control for unspecific binding through the FtsZΔC constructs. Using our assay we can specifically pull down FtsZ binding partners.

To validate our pull-down strategy we performed pull-downs with the C-terminus of *B. subtilis* FtsZ on lysates of *E. coli* cultures expressing three *B. subtilis* proteins that have previously been reported to bind to the FtsZ C-terminus, EzrA [25], SepF [26] and MinC. It has to be noted that evidence for MinC binding to the FtsZ C-terminus comes from the homologous *E. coli* proteins [23]. Although binding for each protein could be expected, we only managed to pull down SepF. Although genetic evidence suggested that MinC_Bs_ needs MinD_Bs_ to act on FtsZ_Bs_ [32] our earlier work showed that MinC_Bs_ alone destabilizes bundles of FtsZ_Bs_ polymers and increases the light scattering signal of non-polymerized FtsZ_Bs_ in solution, indicating that FtsZ_Bs_ and MinC_Bs_ interact without MinD_Bs_ [4]. However, we had not been able to detect interactions between full length MinC_Bs_ and FtsZ_Bs_ through a cross-linking approach [31], whereas MinC_Ec_ cosediments with FtsZ_Ec_ polymers [3] and polymerized FtsZ_Ec_ can be recruited to MinC_Ec_/MinD_Ec_ coated vesicles in a FtsZ_Ec_-C-terminus dependent manner [23]. This suggests that the interaction between *B. subtilis* FtsZ and MinC is not as strong as between the *E. coli* proteins. We do not know why we have not been able to pull down EzrA, as previously a peptide corresponding to the C-terminal 17 amino acids of FtsZ was shown to competitively inhibit EzrA binding to FtsZ and bound to EzrA with a dissociation constant of 102 μM [25]. The interaction between FtsZ and EzrA is sensitive to increasing salt concentrations [25] but our pull-down assays were performed at 150 mM NaCl, a salt concentration at which at least 50% of binding should still occur. Possibly we need a higher expression of His-EzrA_cyt_ or to perform the pull-down assay under cross-linking conditions, or both, to detect the interaction between EzrA and the FtsZ C-terminus.

We have shown that our pull-down assay is very specific. Only SepF was pulled down from an *E. coli* lysate and control experiments showed that this pull-down was dependent on the presence of the C-terminus and independent of the tag attached to SepF. Furthermore, we could not detect SepF mutants that have lost the capability to bind FtsZ and we could show that FtsZ recovery in the reverse pull-down experiment depended on the presence of the FtsZ C-terminus (Fig 2). Our results strongly suggest that the FtsZ C-terminus is the only binding site for SepF. In an earlier study SepF has been found to co-precipitate with FtsZΔC16 polymers, which led the authors to suggest a potential secondary binding site for SepF on FtsZ [26]. We attribute the observed co-sedimentation of SepF with FtsZΔC16 polymers [26] to the buffer conditions used by the authors – 25 mM Pipes at pH 6.8. In an earlier report we have shown that SepF forms rings at pH 7.5 and high salt, but precipitates as aggregates at pH <7 [18]. *In vitro* polymerization and tubulation experiments confirmed that the formation of FtsZ-SepF tubules depends on the presence of the FtsZ C-terminus (Fig 3). SepF sedimentation is dependent on the presence of FtsZ polymers, and does not occur when we use polymers of FtsZ_P372A_ or SepF alone (Fig. 3G).

To identify residues in the FtsZ C-terminus involved in SepF binding we performed an alanine-scan of the C-terminus and performed pull-down assays. Mutation of the two residues that are totally conserved across species, Pro372 and Phe374, abolished the FtsZ-SepF interaction. In total, 11 out of the 16 mutations tested considerably affected SepF binding to the C-terminus. This suggests that the secondary and teriary structure of the FtsZ C-terminus is recognized by SepF, in a way that is more similar to how ZipA binds FtsZ, through hydrophobic contacts and hydrogen bonds to the peptide backbone, rather than to how FtsA binds FtsZ, through specific salt bridges [22], [29]. We cannot exclude that our mutations alter the structure of the C-terminus in such a way that residues are no longer in the right conformation to form salt bridges – for that structural information is required. Recently, the FtsZ C-terminus was divided into a conserved C-terminal tail (CTT) that is followed by a highly variable – both in length and composition – set of residues, the C-terminal Variable region (CTV) [27]. The CTT is thought to constitute the ‘landing pad’ for FtsZ-binding proteins whereas the CTV plays a role in promoting lateral interactions between FtsZ polymers [27]. The *B. subtilis* FtsZ CTV comprises residues 377–382. We noted that several residues in the CTV are likely to play a role in SepF binding, as alanine mutations of residues 377, 378, 380 and 381 led to a considerable decrease in SepF binding. Also, the salt bridge between the C-terminal residue of *T. maritima* FtsZ, which has a 7 residue long CTV, and FtsA [29], suggests that the CTV is part of the ‘landing pad’ for interacting proteins, next to its role in promoting lateral interactions. However, the finding that the *E. coli* FtsZ C-terminus, with a different and noticeably shorter CTV, also binds SepF, supports the notion that the CTT is the main binding partner for FtsZ-binding proteins. More information on the interactions between the FtsZ C-terminus and several FtsZ-binding proteins is required to elucidate this question.

In conclusion, we have developed a novel and highly specific assay to identify interaction partners of the C-terminus of FtsZ from *B. subtilis* and *E. coli*, and validated the assay with candidate binding proteins. Our results show that SepF specifically binds the C-terminus whereas the interactions of *B. subtilis* MinC and EzrA are too weak to be detected in our assay. We are currently using this assay to look for novel proteins interacting with the FtsZ C-termini from lysates of *B. subtilis* and *E. coli* cultures.

## Materials and Methods

### Plasmid construction

All plasmids are listed in Table 1. The sequence coding for the C-terminal 67 amino acid residues (317–383) from *E. coli ftsZ* was amplified from *E. coli* K12 chromosomal DNA by PCR using primers djs414 and djs415 (primers listed in Table 2). The sequence coding for the C-terminal 69 amino acid residues (314–382) from *B. subtilis ftsZ* was amplified from *B. subtilis* 168 chromosomal DNA by PCR using primers djs416 and djs417. Both PCR products were digested with *Pme*I and *Sgf*I and ligated into *Pme*I/*Sgf*I digested pFN18A (Promega), resulting in plasmids pDJ64 (*E. coli ftsZ*) and pDJ65 (*B. subtilis ftsZ*). pDJ65 was used as a template for site directed mutagenesis according to the Quickchange protocol (Stratagene), to introduce a stop codon at the position of D367, and to systematically change residues 367 to 382 to alanine, using primers djs426 to djs459. Similarly, pDJ64 was used as a template for site directed mutagenesis, to introduce a stop codon at the position of D370, and to change residues P375 to alanine, using primers djs461 to djs464. The sequence coding for the soluble part of *B. subtilis* EzrA (amino acid residues 27–562) was amplified from *B. subtilis* 168 chromosomal DNA by PCR using primers ek13 and ek14. The PCR product was digested with *NcoI* and *XbaI* and ligated into *NcoI*/*XbaI* digested pET302, resulting in plasmid pEK5. All constructs were verified by sequencing.

**Table 1:** Plasmids.

*Plasmids*	*Relevant characteristics*	*Source*
pFN18A HaloTag® T7 Flexi® Vector	*bla P_T7_ HaloTag-TEVsite*	Promega
pET302	*lacZα, trc HisTag-enterokinase site*	[34]
pDJ15	*tetR PtetA-minC-strep-tagII*	[4]
pDJ26	*bla lacI^Q^ P_T7_-his_8_-zapA*	[4]
pMal-SepF	*bla P_tac_ malE-sepF*	[18]
pMal-SepF-A98V	*bla P_tac_ malE-sepF_A98V_*	[18]
pMal-SepF-F124S	*bla P_tac_ malE-sepF_F124S_*	[18]
pDJ64	*bla P_T7_ HaloTag-TEVsite-ftsZ_Ec317-383_*; ZC_Ec_	this work
pDJ65	*bla P_T7_ HaloTag-TEVsite-ftsZ_Bs314-382_*; ZC	this work
pDJ69	*bla P_T7_ HaloTag-TEVsite-ftsZ_Bs314-366_*; ZΔC16	this work
pDJ73	*bla P_T7_ HaloTag-TEVsite-ftsZ_Ec317-369_*; Z_Ec_ΔC14	this work
pDJ74	*bla P_T7_ HaloTag-TEVsite-ftsZ_Ec317-P375A-383_*; ZC_EcP375A_	this work
pDJ367-382	*bla P_T7_ HaloTag-TEVsite-ftsZ_Bs314-382_*; residue in plasmid name changed to Ala	this work
pEK5	*lacZα, trc HisTag-enterokinase site- ezrA_27-562_*	this work

**Table 2:** Primers.

primer name	primer sequence 5′-3′	aa change
djs414	GTCCGCGATCGCCATGGACAAACGTCCTG	
djs415	CAGCGTTTAAACTTAATCAGCTTGCTTACG	
djs416	GTCCGCGATCGCCACCGGCTTTATCGAAC	
djs417	GAGCGTTTAAACTTAGCCGCGTTTATTACGG	
djs426	CTTCACAGCCGGCTGATTAAACGCTTGACATCCCGAC	D367Stop
djs427	GTCGGGATGTCAAGCGTTTAATCAGCCGGCTGTGAAG	D367Stop
djs428	GATGATACGCTTGACATCGCGACATTCTTAAGAAACC	P372A
djs429	GGTTTCTTAAGAATGTCGCGATGTCAAGCGTATCATC	P372A
djs430	GAAACCGTAATAAACGCGCCTAAGTTTAAACGAATTC	G382A
djs431	GAATTCGTTTAAACTTAGGCGCGTTTATTACGGTTTC	G382A
djs432	CTTAAGAAACCGTAATAAAGCCGGCTAAGTTTAAACG	R381A
djs433	CGTTTAAACTTAGCCGGCTTTATTACGGTTTCTTAAG	R381A
djs434	CATTCTTAAGAAACCGTAATGCACGCGGCTAAGTTTAAACG	K380A
djs435	CGTTTAAACTTAGCCGCGTGCATTACGGTTTCTTAAGAATG	K380A
djs436	CGACATTCTTAAGAAACCGTGCTAAACGCGGCTAAGTTTAAAC	N379A
djs437	GTTTAAACTTAGCCGCGTTTAGCACGGTTTCTTAAGAATGTCG	N379A
djs438	CCGACATTCTTAAGAAACGCTAATAAACGCGGCTAAG	R378A
djs439	CTTAGCCGCGTTTATTAGCGTTTCTTAAGAATGTCGG	R378A
djs440	CCCGACATTCTTAAGAGCCCGTAATAAACGCGG	N377A
djs441	CCGCGTTTATTACGGGCTCTTAAGAATGTCGGG	N377A
djs442	CATCCCGACATTCTTAGCAAACCGTAATAAACGCG	R376A
djs443	CGCGTTTATTACGGTTTGCTAAGAATGTCGGGATG	R376A
djs444	CTTGACATCCCGACATTCGCAAGAAACCGTAATAAAC	L375A
djs445	GTTTATTACGGTTTCTTGCGAATGTCGGGATGTCAAG	L375A
djs446	GATACGCTTGACATCCCGACAGCCTTAAGAAACCGTAATAAAC	F374A
djs447	GTTTATTACGGTTTCTTAAGGCTGTCGGGATGTCAAGCGTATC	F374A
djs448	CGCTTGACATCCCGGCATTCTTAAGAAACC	T373A
djs449	GGTTTCTTAAGAATGCCGGGATGTCAAGCG	T373A
djs450	GCTGATGATACGCTTGACGCCCCGACATTCTTAAGAAAC	I371A
djs451	GTTTCTTAAGAATGTCGGGGCGTCAAGCGTATCATCAGC	I371A
djs452	GGCTGATGATACGCTTGCCATCCCGACATTCTTAAG	D370A
djs453	CTTAAGAATGTCGGGATGGCAAGCGTATCATCAGCC	D370A
djs454	CCGGCTGATGATACGGCTGACATCCCGACATTC	L369A
djs455	GAATGTCGGGATGTCAGCCGTATCATCAGCCGG	L369A
djs456	CCGGCTGATGATGCGCTTGACATCC	T368A
djs457	GGATGTCAAGCGCATCATCAGCCGG	T368A
djs458	CAGCCGGCTGATGCTACGCTTGACATC	D367A
djs459	GATGTCAAGCGTAGCATCAGCCGGCTG	D367A
djs461	CAAACTGCGAAAGAGCCGTAATATCTGGATATCCCAGC	FtsZ_Ec_D370Stop
djs462	GCTGGGATATCCAGATATTACGGCTCTTTCGCAGTTTG	FtsZ_Ec_D370Stop
djs463	GGATTATCTGGATATCGCAGCATTCCTGCGTAAG	FtsZ_Ec_P375A
djs464	CTTACGCAGGAATGCTGCGATATCCAGATAATCC	FtsZ_Ec_P375A
ek13	AGACTACCATGGCCGAAATCGACCGGCTGGA	
ek14	CGTTACTCTAGACTAAGCGGATATGTCAGCTTTG	

### Protein purification

MBP-SepF and SepF were purified as described [18]. FtsZ was purified as described [4], FtsZΔC16 and FtsZ_P372A_ were purified using the same protocol.

### Pull down experiments

Expression of HaloTagged constructs was done in *E. coli* BL21(DE3) carrying plasmid pBS58 which encodes an extra copy of *ftsQAZ* [33]. Freshly transformed cells were grown overnight at 37°C, diluted 1∶100 and grown to an OD_600_ ∼0.7 and induced with 1 mM IPTG. After three hours of growth cells were harvested by centrifugation and washed with PBS. Cell pellets were flash frozen in liquid nitrogen and stored at −20°C. To prepare lysates, cells were taken up in 1/10^th^ of the original volume in buffer HPB (50 mM HEPES pH 7,5; 150 mM NaCl). Lysozyme (0,1 mg/ml) and DNAse (1 µg/ml) were added and the suspensions were incubated for 10 min on ice. Cells were disrupted using sonication (12 cycles of 5s with 5s cooling at 10 microns probe amplitude). Aliquots of 1ml were made and stored at −20°C. For a typical pull down experiment, for every construct tested a 1 ml aliquot was thawed on ice and subsequently centrifuged at 10000×*g* for 30 min at 4°C. DTT (to 1 mM) and Nonidet P-40 (to 0,005% v/v) were added to the supernatant. The rest of the procedure was carried out in buffer HPB+ (50 mM HEPES pH 7,5; 150 mM NaCl; 1 mM DTT; 0,005% v/v nonidet P-40). To the supernatant, 200 µl (25%) of HaloLink resin (Promega) equilibrated with HPB+ was added. The samples were incubated for 1 h at RT with gentle mixing. Subsequently, the samples were centrifuged at 1000×*g* for 5 min at RT. The supernatant was discarded and the resin was washed 3 times with HPB+. The resin with bound HaloTagged bait protein were subsequently incubated with 1 mL cell lysates (10× concentrated with respect to the original culture volume) of *E. coli* strains overexpressing potential binding partners. Alternatively, the resin with bound Halo-tagged bait protein was incubated with 1 mL of MBP-SepF, MBP-SepF_A98V_, MBP-SepF_F124S_ or SepF at 15 μM diluted in HPB+. For the alanine-scanning experiments, 300 μL of 30 μM SepF was used. The samples were incubated for 1 h at RT with gentle mixing. Subsequently, the samples were centrifuged at 1000×*g* for 5 min at RT, and the supernatant was kept as the flow-through fraction. The resin was washed 3 times with HPB+. The HaloTagged bait protein and associated proteins were released from the resin by TEV-cleavage using 50 μL of Cleavage solution (HPB+ containing TEV-protease, Promega) for 1 h at RT with gentle mixing. The eluted material was collected by centrifugation at 1000×*g* for 5 min at RT and the resin was washed with an additional 50 μL of HPB+ which was collected by centrifugation and added to the eluted material. Samples were prepared for SDS-PAGE and run on 15% gels, which were stained with Coomassie Brilliant Blue.

For the FtsZ pull-downs with MBP-SepF, MBP-SepF bound to amylose-resin was prepared as described [18]. 250 μL aliquots of amylose resin with MBP-SepF were incubated with 300 μL of 30 μM FtsZ, FtsZΔC16 and FtsZ_P372A_ in buffer A (20 mM Tris–HCl pH 7.4, 200 mM KCl, 1 mM EDTA). The proteins were allowed to bind to the resin for 30 min at 4°C. Unbound protein and liquid was removed by mild centrifugation and collected as Flow-through. The resin was washed 6 times with buffer A and the last wash fraction was collected. MBP-SepF and bound FtsZ were eluted from the resin with 300 μL of 10 mM maltose in buffer A. Equal amounts of the input material, the flow-through, last wash and eluate were loaded on 12% SDS-PAGE gels, which were either stained with Coomassie Brilliant Blue, or subsequently transferred to PVDF using Western Blotting and developed using anti-FtsZ.

### Electron microscopy

FtsZ, FtsZ_P372A_, FtsZΔC16 (at 10 µM) and SepF (6 µM) were incubated in polymerization buffer (50 mM HEPES/NaOH, pH = 7,4, 300 mM KCl, 10 mM MgCl_2_) for 5 min. at 30°C. GTP was added to a final concentration of 2 mM to start FtsZ polymerization and the samples were incubated for 20 min. at 30°C. 2 µl of each sample was applied onto a glow-discharged 400 mesh carbon-coated copper grid, incubated for 30 s., blotted dry with filter paper and negatively stained using 5µl uranyl-acetate (2%). The grids were viewed in a Philips CM120 electron microscope equipped with a LaB_6_ filament operating at 120 kV. Images were recorded with a Gatan 4000 SP 4 K slow-scan CCD camera at 36,400× magnification.

### Sedimentation of tubules

FtsZ or FtsZ_P372A_ (at 10µM) was mixed with SepF (12 µM) in polymerization buffer and incubated for 5 minutes at 30°C. Polymerization was started by the addition of GTP to a final concentration of 2 mM, and samples were incubated for a further 20 min. at 30°C. Control samples contained only FtsZ, FtsZ_P372A_ or SepF. Tubules were spun down for 15 min. at 24,600×*g*, at 25°C. Pellet and supernatant fractions were recovered and equal amounts were analyzed by SDS-PAGE and staining with Coomassie Brilliant Blue.
